# Host Cell Death Responses to Non-typhoidal *Salmonella* Infection

**DOI:** 10.3389/fimmu.2019.01758

**Published:** 2019-07-26

**Authors:** Madeleine A. Wemyss, Jaclyn S. Pearson

**Affiliations:** ^1^Department of Molecular and Translational Research, Monash University, Clayton, VIC, Australia; ^2^Department of Microbiology, Monash University, Clayton, VIC, Australia

**Keywords:** non-typhoidal *Salmonella*, programmed cell death, innate immunity, T3SS effector protein, immune evasion, host-pathogen interaction

## Abstract

*Salmonella enterica* subsp. *enterica* serovar Typhimurium (*S*. Typhimurium) is a Gram-negative bacterium with a broad host range that causes non-typhoidal salmonellosis in humans. *S*. Typhimurium infects epithelial cells and macrophages in the small intestine where it replicates in a specialized intracellular niche called the *Salmonella*-containing vacuole (SCV) and promotes inflammation of the mucosa to induce typically self-limiting gastroenteritis. Virulence and spread of the bacterium is determined in part by the host individual's ability to limit the infection through innate immune responses at the gastrointestinal mucosa, including programmed cell death. *S*. Typhimurium however, has evolved a myriad of mechanisms to counteract or exploit host responses through the use of Type III Secretion Systems (T3SS), which allow the translocation of virulence (effector) proteins into the host cell for the benefit of optimal bacterial replication and dissemination. T3SS effectors have been found to interact with apoptotic, necroptotic, and pyroptotic cell death cascades, interfering with both efficient clearance of the bacteria and the recruitment of neutrophils or dendritic cells to the area of infection. The interplay of host inflammation, programmed cell death responses, and bacterial defenses in the context of non-typhoidal *Salmonella* (NTS) infection is a continuing area of interest within the field, and as such has been reviewed here.

## Pathogenicity and Virulence of *Salmonella enterica* Serovar Typhimurium

Infections caused by *Salmonella enterica* are a major challenge in both human and animal health. *Salmonella enterica* subsp*. enterica* serovars are categorized by their disease phenotypes into typhoidal (Typhi and Paratyphi) and non-typhoidal *Salmonella* (NTS) serovars (e.g., *S*. Typhimurium). Whereas, typhoidal serovars cause invasive disease and are human restricted, NTS serovars cause disease in a wide range of mammals and birds and typically cause self-limiting gastroenteritis (salmonellosis) in humans, with the bacteria restricted to the gastrointestinal mucosa (1, 2). *S*. Typhimurium is acquired via the fecal-oral route from consumption of raw or contaminated poultry products, and causes the majority of notified NTS infections in Australia (3, 4). In immunocompromised individuals, *S*. Typhimurium can cause invasive disease that requires antibiotic treatment or hospitalization. Murine infections with *S*. Typhimurium result in invasive disease and bacteremia, and thus are a more representative model of invasive salmonellosis but are nevertheless used to great effect to study the pathogenesis of *S*. Typhimurium *in vivo*.

**Figure 1:**
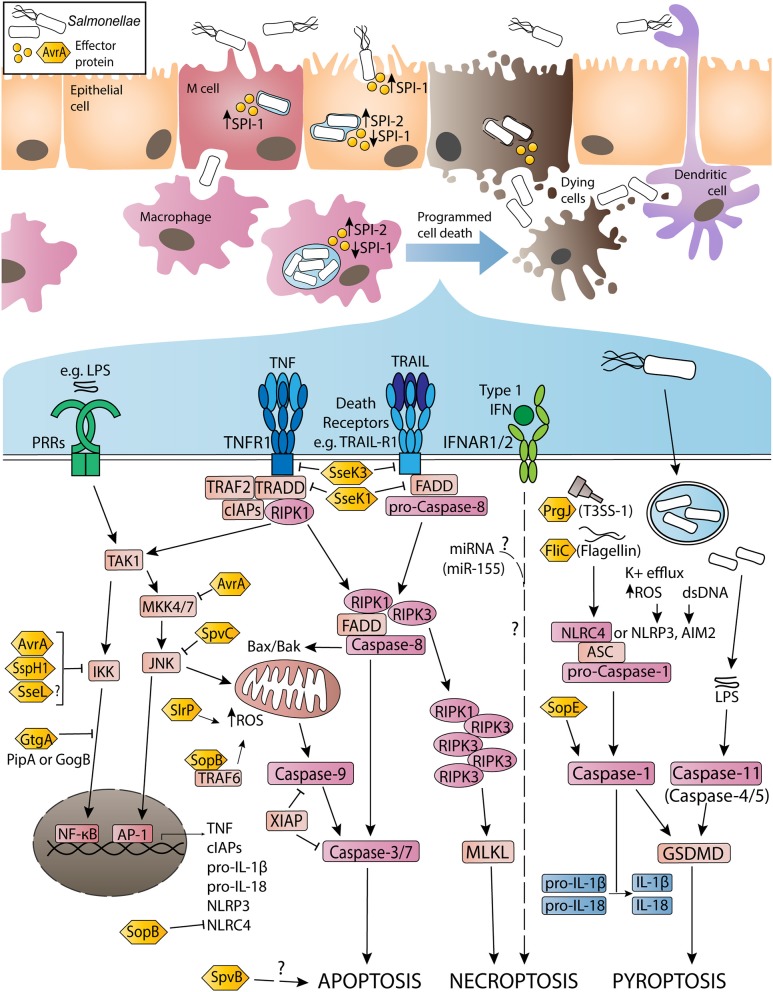
Activation and inhibition of apoptosis, necroptosis and pyroptosis by non-typhoidal *Salmonella* virulence (effector) proteins and other stimuli during *Salmonella enterica* serovar Typhimurium infection. Non-typhoidal *Salmonella* species invade intestinal epithelial cells through the use of SPI-1 effectors to induce membrane ruffling and actin rearrangement, resulting in non-phagocytic uptake of the bacteria. Alternatively, *Salmonella* uptake can occur due to M cell mediated transport across the epithelial barrier, or through sampling by phagocytic cells such as dendritic cells or macrophages. Once internalized, the SPI-1 T3SS and effectors are downregulated, while SPI-2 is upregulated to promote SCV formation and facilitate *Salmonella* replication. Throughout the infection, both SPI-1 and SPI-2 effector proteins interact with host innate immune pathways to either activate or inhibit inflammatory responses and programmed cell death. Signaling cascades have been simplified for clarity and are discussed in more detail in-text.

The interactions between NTS and host cell processes during host invasion and the initial establishment of infection have been reviewed previously by LaRock et al. and as such are only briefly described here (5). Once ingested, *S*. Typhimurium enters the gastrointestinal tract and uses flagella to access the epithelial layer of the terminal ileum. Inflammatory responses in the epithelium release key nutrients required by the bacteria, also causing diarrheal symptoms that promote transmission (6, 7). Following contact with the epithelium, *S*. Typhimurium utilizes a Type III Secretion System (T3SS) encoded on *Salmonella* pathogenicity island-1 (SPI-1) to translocate effector proteins (such as SopE2, SipA, and SopB) into the epithelial cell cytosol, inducing actin rearrangement, membrane ruffling, and non-phagocytic cellular uptake of the bacteria into the host cell (Figure 1) (8–10). Inside the intracellular space, flagella are no longer required for motility of the bacteria, and are typically downregulated in order to avoid host immune responses (11–14). Internalization of *S*. Typhimurium causes formation of an endosome termed the early *Salmonella*-containing vacuole (SCV). Here, a second T3SS (encoded by the SPI-2 locus) is used to translocate virulence proteins such as SifA, SopD2, and SseJ, acidifying the vacuole and maturing the SCV into the ideal replicative niche for the bacteria (15). The late stage SCV enables efficient bacterial replication, while interconnected networks of *Salmonella*-induced filaments (SIFs) allow enclosed bacteria to acquire nutrients (15, 16). Other SPI-2 effectors prevent lysosomal fusion with the SCV, inhibiting recruitment of lysosomal enzymes and trafficking markers that would promote degradation of the vacuole (15, 17). In epithelial cells, subpopulations of *S*. Typhimurium have been observed in the cytosol, resulting in bacterial hyper-replication and host cell extrusion (18). Host guanylate-binding proteins (GBPs), expressed following Type I or II interferon (IFN) signaling, can also lyse the SCV, exposing *S*. Typhimurium to the cytosol (19–21).

Cytosolic *S*. Typhimurium enable the detection of pathogen-associated molecular patterns (PAMPs), such as lipopolysaccharide (LPS) and flagellin, by pattern recognition receptors (PRRs) or Nod-like receptors (NLRs). PRRs act to recruit immune cells to infected tissues and limit bacterial virulence through the activation of pro-inflammatory signaling. The ability of *Salmonella* infection to induce tumor necrosis factor (TNF) production in epithelial cells and macrophages is well-documented (22–24). TNF signaling typically reinforces the production of pro-survival cytokines and anti-apoptotic factors via nuclear factor kappa B (NF-κB) or mitogen-activated protein kinase (MAPK) signaling cascades (25–27). However, effector proteins such as GtgA, SspH1, SptP, and potentially SseL can prevent the activation of these pathways, instead driving TNF signaling toward programmed cell death cascades (28–34). These include apoptosis, necroptosis, and pyroptosis, and are triggered by TNF and other death receptor ligands, or inflammasome activation (Figure 1). Death of the host cell allows escape of *S*. Typhimurium into the extracellular space, and uptake of the bacteria by professional phagocytes. Neutrophils play a key role in the overall clearance of *S*. Typhimurium, killing the phagocytosed bacteria through the activity of reactive oxygen species (ROS), while infected dendritic cells and macrophages can spread the bacteria to the mesenteric lymph nodes, spleen, and liver (12, 35–37).

## Apoptotic Cell Death Pathways During *Salmonella* Infection

Apoptosis is a caspase-dependent form of programmed cell death, induced in damaged or stressed cells in response to intrinsic or extrinsic signaling cascades (38). The apoptotic process results in DNA fragmentation, phosphatidylserine exposure, formation of apoptotic bodies, and the display of “eat me” signals to prompt phagocytic clearance of the dying cell. Intrinsic apoptosis is triggered by DNA damage, accumulation of ROS or endoplasmic reticulum (ER) stress, resulting in mitochondrial outer membrane permeabilization and activation of caspase-9. Caspase-9 catalyzes the activation of caspase-3 and caspase-7, which execute the biochemical and morphological changes characteristic of apoptosis (38). In contrast, extrinsic apoptosis responds to ligand or cytokine binding to transmembrane death receptors on the surface of the cell. Death receptors possess an apoptosis-activating death domain, and include receptors such as Fas, TNFR1, and TRAIL-R1. Upon TNF stimulation, TNFR1 recruits adaptor proteins such as TNFR1-associated death domain protein (TRADD), TNFR-associated factor 2 (TRAF2) and receptor-interacting serine/threonine-protein kinase 1 (RIPK1) (Figure 1). RIPK1 is subject to ubiquitylation and phosphorylation events that direct TNFR1 signaling toward pro-survival NF-κB activation (39). In the absence of modifications, RIPK1 associates with pro-caspase-8, TRADD and Fas-associated protein with death domain (FADD) to form a cytosolic secondary signaling complex (40, 41). Cellular FLICE-like inhibitory protein (cFLIP) also regulates complex assembly by inhibiting caspase-8 activation (42). Secondary complex activation allows caspase-8 to activate caspase-3/-7 and subsequent apoptosis.

During *S*. Typhimurium infection, autocrine or paracrine TNF signaling triggers cell death responses by initiating extrinsic apoptosis. *Salmonella* effector proteins also induce apoptosis via these signaling pathways. SlrP is an E3 ubiquitin ligase translocated by both SPI-1 and SPI-2 that interacts with thioredoxin-1 (Trx1) and the ER chaperone protein, ERdj3 (43–46). Expression of SlrP increased cytotoxicity in infected HeLa cells, suggesting a role for SlrP in inducing intrinsic apoptosis in infected epithelial cells (43, 44). Additionally, translocation of SPI-2 effector SpvB (an ADP-ribosylase) promotes apoptosis in human monocyte-derived macrophages (HMDMs), potentially due to loss of polymerized F-actin (47–50). SpvB may have a similar effect in *S*. Dublin-infected HT-29 cells, although apoptosis was markedly delayed in these cells (28 h post-infection *in vitro*) (51). However, the mechanism by which SpvB promotes apoptosis remains unclear.

Alternatively, effectors such as SopB may have a role in preventing intrinsic apoptosis. SopB (also known as SigD) is a phosphoinositide phosphatase translocated by SPI-1 that has multiple reported virulence functions (8, 52, 53). Infection of mouse embryonic fibroblasts revealed that SopB is ubiquitylated by TRAF6, potentially as a mechanism of directing SopB activity within the host cell (54, 55). SopB-TRAF6 interactions prevent the recruitment of TRAF6 to the mitochondria, inhibiting accumulation of ROS in the organelle, thus preventing intrinsic apoptosis (56). SopB phosphatase activity in epithelial cells also mediates the recruitment of Rho and Ras family GTPases to the site of infection, promoting pro-survival Akt signaling and inhibiting apoptotic responses downstream (57–59). Another SPI-1 translocated effector that alters apoptotic pathways is AvrA, which displays deubiquitinase and acetyl-transferase activity. Studies of *S*. Typhimurium-infected HeLa or HCT116 cells demonstrated that AvrA deubiquitylates IκBα to suppress pro-survival NF-κB activation (60, 61). Interestingly, *in vivo* mouse infections, as well as transfection of AvrA into HEK293T cells, indicated that AvrA also prevents apoptotic responses by acetylating MAPK kinase 4 (MKK4) and inhibiting the c-Jun N-terminal-kinase (JNK) pathway (62–64). Similarly, SpvC (a phosphothreonine lyase) acts to both dampen inflammation and suppress apoptosis by inactivating members of the MAPK pathway (65, 66). While suppression of both pro-survival signaling and pro-apoptotic pathways may initially seem counterintuitive, it is likely that this duality allows *Salmonella* to prolong infection of epithelial cells, allowing greater opportunities for replication in this cell type.

## *Salmonella* Infection and Necroptotic Cell Death

Necroptosis is a caspase-independent lytic form of programmed cell death that results in characteristic pore formation and the release of cellular contents and highly inflammatory damage-associated molecular patterns (DAMPs) into the extracellular space (67). Initially triggered by TNF binding to TNFR1, necroptosis occurs when caspase-8 is non-functional or inhibited. In the absence of active caspase-8, deubiquitylated RIPK1 is able to interact with RIPK3, subsequently forming an amyloid-like complex (called the necrosome), activating RIPK3 via autophosphorylation events (68). Active RIPK3 then mediates the phosphorylation of mixed lineage kinase domain-like protein (MLKL), enabling MLKL oligomerization and migration to the plasma membrane, triggering membrane permeabilization and lytic cell death (Figure 1) (68). Although RIPK3 and MLKL are critical for the induction of necroptosis, the precise mechanism by which active MLKL executes necroptosis remains unclear (38, 69, 70). Released DAMPs induce inflammatory responses in neighboring cells, promoting recruitment of innate immune cells and mediating tissue pathology in the immediate area (71).

Observations of necroptosis in response to *S*. Typhimurium infection have included studies comparing infected C57BL/6J wild type (WT) or type I IFN alpha/beta receptor 1 deficient (*Ifnar1*^−/−^) mice (72). Type I IFNs act through heterodimeric IFNAR1/IFNAR2 complexes to activate Janus kinase (JAK)/signal transducer and activator of transcription (STAT) signaling cascades, resulting in the transcription of interferon-stimulated genes (ISGs) (73). Following intravenous *S*. Typhimurium infection, *Ifnar1*^−/−^ mice experienced improved survival compared to WT mice, while infected *Ifnar1*^−/−^ bone marrow derived macrophages (BMDMs) experienced reduced rates of cytotoxicity *in vitro*, with decreased activation of RIPK1 and RIPK3 (72). Immunoprecipitation of IFNAR1 in WT BMDMs indicated RIPK1 associates with IFNAR1 following Type I IFN stimulation, while *in vivo* infection of *Ripk3*^−/−^ mice induced similar cytotoxicity to *Ifnar1*^−/−^ mice (72). Robinson et al. thus proposed a role for Type I IFN signaling in inducing necroptosis in *S*. Typhimurium-infected macrophages (72). Later work found that signaling downstream of IFNAR1/RIPK1/RIPK3 interactions resulted in recruitment of phosphoglycerate mutase family member 5 (PGAM5) (74). PGAM5 recruitment by RIPK3 was suggested as a mechanism of promoting or executing necroptosis in *S*. Typhimurium-infected BMDMs via impaired production of antioxidants, resulting in ROS-mediated mitochondrial damage (74, 75). However, studies outside the *S*. Typhimurium infection context did not support PGAM5 as a mediator of necroptosis, instead proposing that PGAM5 counteracts necroptosis by promoting autophagic degradation of mitochondria (inhibiting ROS production) (70, 76, 77).

Other explorations of necroptosis in the context of *S*. Typhimurium infection involved the use of qRT-PCR techniques to assess the expression of micro RNAs (miRNAs) induced by infection in RAW264.7 cells (78). A highly upregulated miRNA, miR-155, mediated cytotoxicity levels similar to *S*. Typhimurium-infected cells when transfected into RAW264.7s (78). Further *in vitro* transfections indicated that miR-155 induced RIPK1 and RIPK3 phosphorylation (indicative of necroptosis) by 18 h post-treatment, as well as cleavage of poly (ADP-ribose) polymerase-1 (PARP-1) in a similar manner to *S*. Typhimurium infection (78). Treatment with RIPK1 inhibitor necrostatin-1s partially rescued cell viability in miR-155 transfected cells, supporting a role for necroptosis in contributing to cytotoxicity (78). The authors suggested that PARP-1 activation occurs downstream of RIPK1/RIPK3 activation, however existing work in TNF-stimulated L929 cells instead proposes that PARP-1 contributes to a separate programmed necrosis pathway (78, 79).

Virulence proteins may also play a role in mediating host necroptotic responses during *S*. Typhimurium infection. *Salmonella* secreted effector K1 (SseK1), SseK2, and SseK3 are a family of related virulence proteins with glycosyltransferase activity that share high sequence homology with the Arg-GlcNAc transferase, NleB, found in attaching and effacing (A/E) pathogens (80–82). SseK effectors reportedly inhibit TNF-induced NF-κB signaling and cell death in macrophages, through arginine glycosylation of FADD and TRADD by SseK1 and SseK3, respectively (83). *In vitro* infections of RAW264.7 cells with Δ*sseK123 S*. Typhimurium showed similar levels of caspase-3/-7 activation when compared to WT infection, but resulted in higher levels of MLKL phosphorylation, indicating that SseK1 and SseK3 may specifically inhibit necroptotic cell death (83). Reports of SseK binding targets remain inconclusive, with suggested glycosylation targets for SseK1 including GAPDH, FADD, and TRADD, while SseK2 may glycosylate FADD (83–85). Recently, mass spectrometry-based screens have identified TNFR1 and TRAIL-R as novel glycosylation targets of SseK3, and demonstrated that TRADD is the preferred binding target of SseK1 (85). Although the specific actions of SseK effectors have yet to be confirmed, collectively these results suggest that SseK1 and SseK3 modify TNFR superfamily members as well as TRADD or FADD, thus inhibiting both TNF-mediated NF-κB signaling and cell death via apoptosis or necroptosis.

## Inflammasome Activation and Pyroptotic Cell Death During *Salmonella* Infection

Pyroptosis is a highly inflammatory, caspase-dependent form of lytic cell death characterized by pore formation and release of active IL-1β and IL-18 (86). Originally thought to be a caspase-1 dependent form of apoptosis or necrosis, pyroptosis is an important host defense mechanism against *S*. Typhimurium (87–89). Typically, pyroptosis in *S*. Typhimurium-infected cells is triggered by the sensing of flagellin (FliC and FljB) or PrgJ (a SPI-1 rod protein) by NLR family apoptosis inhibitory proteins (NAIPs), which then interact with NLR family caspase activation and recruitment domain (CARD)-containing protein 4 (NLRC4) to trigger assembly of a multiprotein complex called the NLRC4 inflammasome (90–92). NLRC4 recruits pro-caspase-1 via shared CARD domains, and can also recruit apoptosis-associated speck-like protein containing a CARD domain (ASC), to assemble the inflammasome and induce the proteolytic activation of caspase-1 (Figure 1) (92). Active caspase-1 mediates pyroptosis by cleaving gasdermin-D (GSDMD), producing an N-terminal segment that forms multimeric pores in the cell membrane and releases cellular contents into the extracellular space (93, 94). Caspase-1 also cleaves IL-1β and IL-18 into their active forms, allowing their release through the GSDMD-N pores, or following the process of necrosis or others [as reviewed by Eder et al. (95)] (93, 95–98).

Other sensors capable of inducing pyroptosis via ASC-caspase-1 inflammasomes include NLRP3 (senses K+ efflux or increased ROS), AIM2 (detects cytosolic dsDNA) and pyrin (senses inhibition of RhoA GTPase activity) (99–103). Both NLRP3 and NLRC4 contribute to IL-1β and IL-18 maturation and pyroptosis in *S*. Typhimurium-infected macrophages (104). Activated NLRC4 amplifies caspase-1 activation in infected macrophages by recruiting NLRP3, forming a single inflammasome complex with ASC that mediates pyroptotic responses downstream (104–106). Alternatively, non-canonical inflammasome pathways can induce pyroptosis through the sensing of cytosolic LPS by murine caspase-11 (or human caspase-4/-5) which cleaves GSDMD independent of caspase-1 activation, however caspase-11 does not cleave IL-1β or IL-18, thus reducing pro-inflammatory cytokine release (Figure 1) (107–112). Both NLRC4 and non-canonical inflammasome activation play a role in epithelial cell responses to infection, and may help reduce bacterial dissemination throughout the intestinal mucosa (108, 113–116).

Crosstalk with caspase-8 and apoptotic pathways can also promote inflammasome activation in *S*. Typhimurium-infected cells. Studies of NLRP3 and NLRC4 interactions during *S*. Typhimurium infection detected IL-1β maturation mediated by ASC-caspase-8 specks, suggesting a role for caspase-8 as an inflammasome effector (105, 117). Other studies have proposed roles for caspase-8 in priming inflammasome activation, or coordinating cleavage of caspase-1 in the absence of NLRP3 or NLRC4 (118). Although not yet demonstrated, effectors such as SlrP, which induce downstream ROS accumulation, could contribute to inflammasome activation and pyroptotic responses due to NLRP3 detection of ROS. However, a study of IL-1β release in a murine *S*. Typhimurium *in vivo* infection context found that SlrP signaling inhibited IL-1β activation, contradicting this idea (119). Aside from SlrP, effectors such as SipB, SopE, or SopB may influence pyroptosis in *S*. Typhimurium-infected macrophage. Following secretion, SipB interacts with SipC to form a translocon pore, facilitating SPI-1 effector translocation into the host cell (120). SipB is reportedly sufficient to induce caspase-1-mediated “apoptosis” and IL-18 maturation in SipB transfected or *S*. Typhimurium-infected dendritic cells and peritoneal macrophages, potentially via direct interactions with caspase-1 (121–123). These results likely indicate pyroptosis, however the mechanisms by which SipB interact with caspase-1 or the inflammasome remain unclear.

*S*. Typhimurium SPI-1 effector SopE is a guanine nucleotide exchange factor that catalyzes the activation of host cell Rho GTPases such as Cdc42 and Rac1 (124). Activation of Rac1 by SopE has been reported to induce caspase-1 activation and IL-1β secretion during *S*. Typhimurium infection of HeLa or RAW264.7 cells, and *in vivo* infection of murine enterocytes (125). SopE-induced caspase-1 activation in macrophages was not due to NLRC4 sensing of flagellin, suggesting an alternative sensor mechanism (126). Other Gram-negative bacteria possess effectors that modify Rho GTPase activity, for instance *Yersinia* spp. effector YopT, which inhibits the activity of RhoA (102, 127). This RhoA inactivation allows assembly of the pyrin inflammasome, resulting in downstream caspase-1 activity and pyroptosis in infected cells (102). This suggests interesting avenues of research for SopE-induced caspase-1 activation; however, pyrin activation has not been observed in response to changes in Rac1 or Cdc42 activity (127). In contrast to SopE, SPI-1 effector SopB plays a role in dampening inflammasome activation. SopB has been associated with the downregulation of NLRC4 in *S*. Typhimurium-infected macrophages and B cells (128–130). NLRC4 depletion was associated with reductions in both IL-1β maturation and cytotoxicity in *S*. Typhimurium-infected B cells, and was determined to be the result of Akt/YAP pathway activation (128, 130). Loss of NLRC4 inhibits the dominant inflammasome involved in the pyroptotic response to *S*. Typhimurium infection, thus allowing the bacteria better opportunities for replication before escaping the host cell.

Lastly, although *S*. Typhimurium effectors both activate and inhibit inflammasome activation, current understandings of these effectors suggest that their translocation is under temporal and spatial control by the bacteria due to their translocating T3SS type. A recent study demonstrated that mutation of the SPI-1 T3SS resulted in decreased HMDM cytotoxicity and IL-1β release, while infection with ΔSPI-2 *S*. Typhimurium induced rapid cell death and IL-1β production in these cells (131). SPI-2 mutation also resulted in increased expression of SPI-1 effectors detectable by NLRC4 (FljB, PrgI, and PrgJ), suggesting that SPI-2 activity helps suppress the translocation of SPI-1 effectors later in infection (131).

## Concluding Remarks

Investigating cell death in the context of *S*. Typhimurium infection has revealed highly complex interactions between host signaling cascades and bacterial virulence effectors. Tightly regulated control of T3SS effector translocation supports bacterial requirements at different infection stages, allowing *S*. Typhimurium to evade or promote cell death responses. Our understanding of *Salmonella*-host interactions is continually evolving, with virulence mechanisms and effector proteins still to be characterized and improved *in vitro* and *in vivo* models for testing hypotheses frequently emerging. While the current literature does not describe immediate applications for exploiting programmed cell death in treatment of salmonellosis, further exploration of NTS virulence factors could help characterize clinical isolates, leading to personalized therapies and improved patient outcomes. Additionally, the high specificity of *Salmonella* effector proteins could prove crucial to the development of novel genome or proteome editing tools (such as the recently described use of effectors from *Shigella flexneri*) (132). Overall, exploration of pathogen-mediated cell death provides crucial insights into how bacteria can mediate survival and dissemination between host cells and can further improve our general understanding of the importance of cell death in counteracting bacterial pathogenesis.

## Author Contributions

MW wrote the initial manuscript and designed the figure. JP and MW edited and revised the manuscript. Both authors read and approved the final manuscript.

### Conflict of Interest Statement

The authors declare that the research was conducted in the absence of any commercial or financial relationships that could be construed as a potential conflict of interest.

## Abbreviations

A/E: attaching and effacing
AIM2: absent in melanoma 2
AP-1: activator protein 1
ASC: apoptosis-associated speck-like CARD-containing protein
AvrA: avirulence gene A
CARD: caspase activation and recruitment domain
cIAP: cellular inhibitor of apoptosis
EHEC: enterohemorrhagic *Escherichia coli*, EPEC, enteropathogenic *E. coli*
FADD: Fas-associated protein with death domain
GBP: guanylate-binding protein
GSDMD: gasdermin D
IFN: interferon
IFNAR1: interferon alpha/beta receptor alpha chain
IL-1β: interleukin 1β
IKK: inhibitor of kappa kinase complex
JNK: c-Jun N-terminal kinase
LUBAC: linear ubiquitin chain assembly complex
MAPK: mitogen activated protein kinase
MLKL: mixed lineage kinase domain-like protein
MKK4: MAPK kinase 4
NF-κB: nuclear factor kappa B
NLR: Nod-like receptor
NLRC4: NLR family CARD domain-containing 4
NLRP3: NLR family pyrin domain-containing protein 3
PAMP: pathogen-associated molecular pattern
PipA: pathogenicity island-encoded protein A
PRR: pattern recognition receptor
RIPK: receptor interacting serine/threonine protein kinase
TAK1: transforming growth factor beta-activated kinase 1
TLR: Toll-like receptor
TNF: tumor necrosis factor
TNFR1: TNF receptor 1
TRADD: TNFR1-associated death domain protein
TRAF: TNFR associated factor
TRAIL: TNF-related apoptosis-inducing ligand
TRAIL-R1: TRAIL receptor 1
SIF: *Salmonella* induced filaments
SlrP: *Salmonella* leucine-rich repeat protein
SopB: *Salmonella* outer protein B
SpvB: *Salmonella* plasmid virulence gene B
SseL: *Salmonella* secreted effector L.
